# Stakeholder acceptance of a robot-assisted social training scenario for autistic children compared to a tablet-computer-based approach

**DOI:** 10.1038/s41598-025-93970-x

**Published:** 2025-04-02

**Authors:** Jonas Frenkel, Simone Kirst, Sandra Naumann, Martina Simon, Julian Sessner, Eileen Roesler, Linda Onnasch, Isabel Dziobek

**Affiliations:** 1 Department of Educational Sciences, University of Potsdam, Karl-Liebknecht-Straße 24/25, 14476 Potsdam, Germany; 2 Science of Intelligence, Research Cluster of Excellence, Marchstraße 23, 10587 Berlin, Germany; 3 Clinical Psychology of Social Interaction, Humboldt-Universität zu Berlin, Unter den Linden 6, 10099 Berlin, Germany; 4 Arbeitsgruppe für Supply Chain Services, Fraunhofer IIS, Nordostpark 84, 90411 Nurnberg, Germany; 5 Institute for Factory Automation and Production Systems, Friedrich-Alexander-Universität Erlangen- Nürnberg, Fürther Str. 246b, 90429 Nürnberg, Germany; 6 Human Factors and Applied Cognition, George Mason University, 4400 University Drive, 3F5, Fairfax, VA 22030 USA; 7 Psychology of Action and Automation, Technische Universität Berlin, Marchstraße 23, 10587 Berlin, Germany; 8 German Center for Mental Health (DZPG), Berlin, Germany

**Keywords:** Psychology, Health care, Therapeutics, Mechanical engineering

## Abstract

Recent studies indicate the potential benefits of robot-assisted therapy (RAT) for children on the autism spectrum (AS), yet acceptance among stakeholders remains unclear due to methodological shortcomings in existing research. This study evaluates stakeholders’ acceptance of a RAT-scenario designed to train emotion recognition and regulation in AS children using Softbank Robotics Pepper, a humanoid robot with an integrated tablet screen, compared to a tablet-based therapy (TBT) control. An online survey of 123 stakeholders (caregivers, professionals, and autistic adults) assessed RAT and TBT using global acceptance, intention to use, and acceptance-related factors (ALMERE model), alongside stakeholders’ concerns. While a larger proportion of stakeholders (58%) showed high global acceptance of RAT, stakeholders preferred TBT across measures and groups (*p* < .001, Wilk’s Λ = 0.595), potentially due to its established familiarity and easier usability. The intention to use RAT was predicted by perceived usefulness, and, to a smaller extent, perceived ease of use, and affinity for technology, independent of stakeholder group. Concerns mainly addressed the expected effort to implement RAT in therapeutic services. Overall, the results highlight stakeholder acceptance and underscore the need to enhance RAT’s perceived usefulness and ease of implementation, suggesting a user-centered design approach for future deployments.

## Introduction

Children on the Autism Spectrum (AS) are often characterized by persistent challenges in social interaction and communication, as well as restricted and repetitive behaviors^1^. These challenges can include difficulties in understanding and expressing socio-emotional signals^2–4^, which can lead to problems in establishing and maintaining relationships^5,6^. Additionally, difficulties in emotion regulation frequently amplify these social consequences, potentially causing co-morbid internalizing symptoms (e.g., depression, anxiety) and externalizing symptoms (e.g., aggression)^7^. Given these multifaceted challenges, early intervention programs focusing on socio-emotional functioning are crucial for improving long-term outcomes and mental health for children on the AS.

In designing interventions, it is crucial to account for the significant variability within the Autism Spectrum, encompassing a wide range of strengths and challenges. Children on the AS may respond differently to various approaches based on their individual preferences, cognitive profiles, and specific needs. Many children with AS benefit from structured, predictable environments, which can make technology-based interventions particularly appealing^8^. Consequently, technology-based approaches such as computer programs or virtual-reality games^9–11^ have been increasingly used as complements to standard autism interventions. These approaches are often designed to foster socio-emotional skills by providing immediate feedback, personalized reinforcement, and teacher or parent support^9,12^ and evidence suggests good effectiveness and acceptance of such approaches (e.g^8,13,14^). Social robots have emerged as a promising addition to these interventions, offering consistent and simplified interactions that can reduce human-associated distress^15–17^, while supporting socio-emotional development^18^.

Although mostly preliminary^16^, positive effects of RAT approaches have been reported by numerous studies^16,19,20^ and evidence suggests that RAT approaches could enhance therapy engagement, motivation, and compliance^14,21^, while their adaptability makes them a valuable tool for addressing the diverse needs within the Autism Spectrum.

However, the practical implementation and usage of RAT in real-life settings requires its future users, thus, autistic children and relevant stakeholders (e.g., autism professionals, caregivers, or teachers) to accept these new approaches first^22–24^. Targeting adult stakeholders might provide important insights into the costs, benefits, and potential risks of RAT before their implementation. Further, since direct assessment of the children’s acceptance levels is often limited due to the children’s age, developmental level, or autism severity^12,25,26^, caregivers might yield in-depth knowledge of the children’s therapeutic and educational needs. It might be further essential to assess a more subjective and experienced-based perspective such as that provided by autistic adults to address the potentially fundamental gap between the perspectives of researchers and autistic children^27^.

Nevertheless, in contrast to research on the acceptance of social robots in different application settings or for other target groups^28–30^, the involvement of relevant stakeholders in acceptance studies on RAT for autistic children is limited^20,31,32^. Moreover, most of the existing stakeholder-focused studies focus on the identification and quantification of factors underlying acceptability^24,33–36^, thereby allowing only limited conclusions about general acceptance levels among stakeholders. In contrast to studies targeting the general population, which show mainly critical opinions towards the use of robots in social contexts (e.g., 60% in a survey by the European Commission^37^), the few existing stakeholder studies on general acceptance and related ethical and social aspects often reported rather positive attitudes^25,31,38,39^. For example, Coeckelbergh and Colleagues^31^ reported that 85% of the 416 participants (caregivers of children on the AS, therapists, teachers, and autism specialists) approved of the use of robots in the context of autism therapy and diagnostics. While these results suggest high levels of acceptance for RATs among stakeholders, the overall consensus of the research, however, is not conclusive, as other findings in stakeholder populations also indicated more cautious or skeptical opinions in addition to a general openness to RATs^32,40–45^.

Whether a new technology is accepted depends on a wide set of specific conditions. For example, while many stakeholders in the studies listed above rejected the idea of RAT systems replacing human therapists, concepts integrating the robot in a supportive capacity received greater approval^31,45^. However, many stakeholder studies assessing RAT acceptance lack concrete and specific information on the specific roles and tasks of the robot during therapy^19,45^ and its physical appearance, which are important predictors of acceptance of RAT^25,31,46,47^. Hence, more studies are needed that analyze the acceptance of more elaborated RAT-approaches, that concretely describe the intended robot and clearly target a specific clinical application setting^32^.

Further, limited first-hand experiences with social robots might potentially lead to substantial bias and distortion of the acceptance ratings, since people may rely on existing social representations, mostly originating from pop-cultural entertainment media^48^. This unfamiliarity can result in substantial novelty effects, that can evoke a range of reactions: While often discussed in terms of initially enhanced engagement and acceptance due to the intrigue and curiosity surrounding new technologies, such as robots, this effect is typically transient^49^. As the initial excitement wanes, sustained interaction might decrease if the technology fails to integrate seamlessly into daily routines or if it does not meet long-term expectations^50^. Moreover, unfamiliar aspects of new technologies may also lead to adverse outcomes and can provoke resistance or skepticism among users, particularly if the perceived complexity or the change from traditional methods is significant^49,51^. This resistance often stems from practical usability concerns and deeper psychological discomfort associated with replacing human roles with robotic functions^31,52,53^.

Finally, recent stakeholder studies on RAT acceptance often lack appropriate control conditions, and thus, conclusions were mostly drawn by interpreting averaged Likert scale scores^54^. However, without comparative means, the interpretability of these numerical values remains limited. Suitable comparison conditions to contextualize RAT acceptability ratings might be computer- or tablet-based therapy (TBT) approaches (see^38^ for a first approach), as their popularity in research and practice is increasing and they were evaluated through a growing number of randomized controlled trials^55^.

**Fig. 1:**
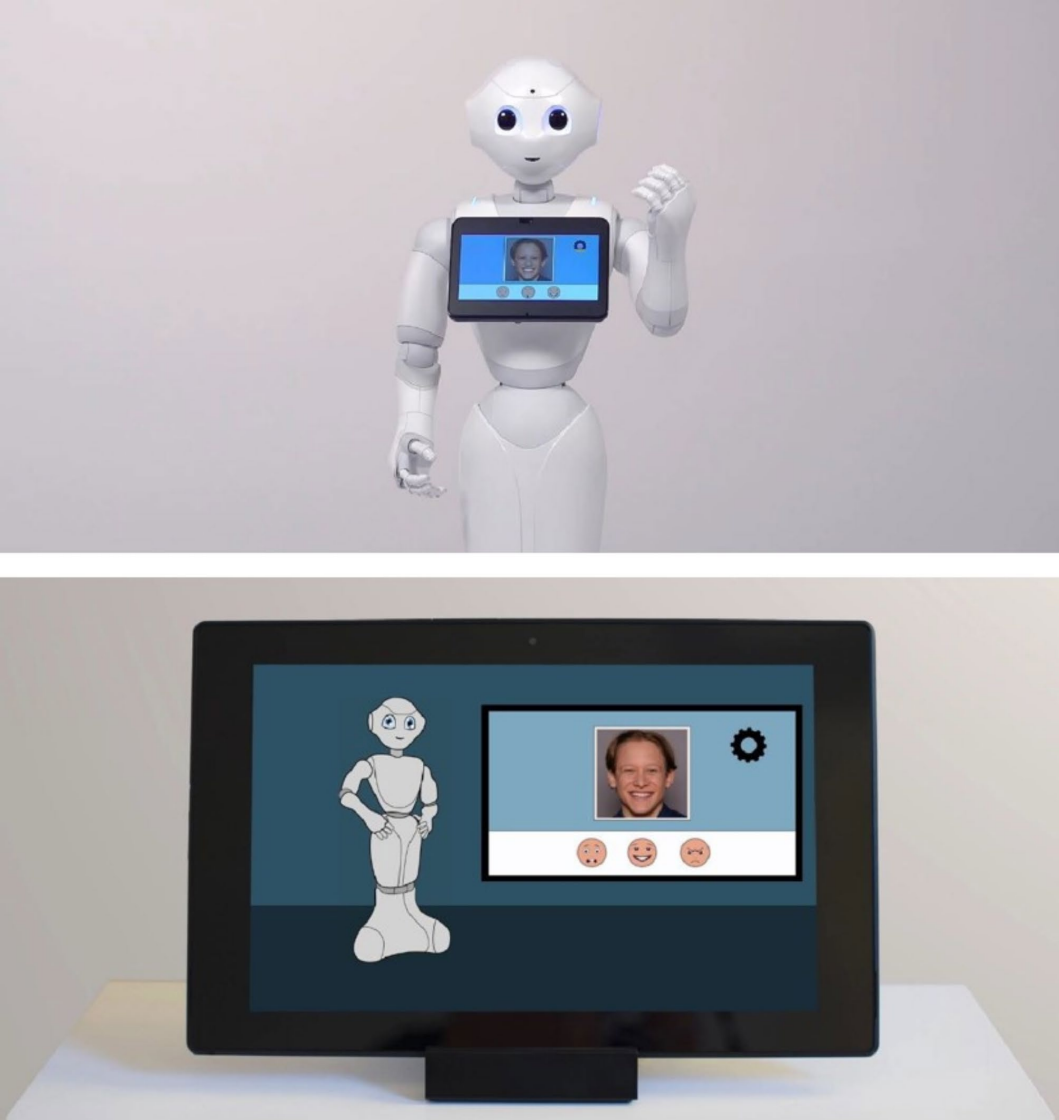
Screenshots of the demonstration videos. (**a**) Robot-assisted training scenario (RAT condition). For the complete video, see: https://www.youtube.com/watch?v=jHW6QCb5H7o (**b**) Tablet-based training scenario (TBT condition). For the complete video, see: https://www.youtube.com/watch?v=6o_uthq8K-Y. *Note*: The image of the human face visible in both conditions is part of a video library designed for training emotion recognition in autistic children. These materials have been used and validated in prior studies, e.g^9,57,58^.

The current study aims to investigate stakeholders’ acceptance of an elaborated RAT-scenario targeted at autistic children.^1^ The training comprises two modules, in which children aged 7–12 engage with the humanoid robot Pepper (Softbank Robotics) (Fig. 1a). The robot acted as a tutor and guided the children through educational games, presented on its’ integrated screen. Module 1 focused on fostering emotion recognition skills, based on videos of facially expressed emotions. Module 2 was aimed at learning how to regulate negative arousal states. To this end, stress/frustration was induced through a video game that involved the child experiencing frequent losses. Throughout the game, the robot regularly instructed the child to do a calming breathing exercise. The robot acted semi-autonomously, with the therapist remaining in supervisory control using an external control tablet^56^. The RAT-scenario was contrasted by a comparable TBT-approach, in which the module contents were transferred to a tablet, with an on-screen virtual avatar assuming the role of the robot (Fig. 1b).

Due to Covid-related restrictions at the time of the study, both conditions (RAT/TBT) were presented via demonstration videos (see Methods section) integrated into an online survey. Potential differences between the evaluations of the target technologies were measured with a single item assessing global acceptance. In addition, we employed a technology acceptance questionnaire, based on the established ALMERE model^22^, to gain more in-depth insights into stakeholders’ attitudes. The questionnaire aims at predicting the behavioral aspect of acceptance, the *intention to use* the technology in its target context, by variables related to functional evaluation (*perceived usefulness*, *perceived ease of use*) as well as social and contextual influencing factors (*social influence*, *attitude towards technology*, *perceived enjoyment*, *facilitating conditions*)^22^. To allow the contribution of a variety of different perspectives, due to stakeholders being anchored in different knowledge systems^59^, we integrated three different stakeholder groups: adults on the AS (*N* = 40), autism professionals (e.g., educators, therapists, *N* = 40), and caregivers of autistic children (*N* = 43), to participate in the survey.

We hypothesized that a larger proportion of participants would show medium to high global acceptance levels as compared to low acceptance levels for the RAT-scenario (H1). In the comprehensive comparison between the technologies and the stakeholder groups, we further expected (H2) that the ratings of all acceptance variables (global acceptance, ALMERE subscales) would differ between the RAT-scenario and the TBT-scenario. No assumption on the direction of the expected effect was made for several reasons: Firstly, the lack of studies directly comparing RAT and TBT-approaches in the field of autism does not provide a sufficient basis for directional hypotheses. Secondly, as acceptance depends on numerous factors that are highly context- and implementation-dependent, a direct transfer of existing findings remains challenging. Thirdly, while tablets represent a more established technology potentially associated with higher acceptance, the novelty effect could influence the acceptance ratings of the RAT-approach in unexpected directions.

In addition to the main analysis, we employed a regression analysis, to predict the *intention to use* the robot in autism therapy by the two variables of the ALMERE model^22^), related to functional evaluation (*perceived ease of use; perceived usefulness*), controlling for the participants’ general affinity for technology. Finally, participants were asked to directly indicate their preference regarding RAT and TBT, and potential concerns associated with RAT or TBT were inquired about to better understand potential acceptance deficits.

## Results

### Participants’ characteristics

Sociodemographic information and clinical characteristics are reported in Table 1. The mean age of the total sample was 41.85 years (*SD* = 10.34). 89 participants were female, 32 male, and two non-binary. Obtaining a university degree was the most frequently indicated educational level across all groups (*N* = 81). On average, autism professionals had *M* = 9.20 years (*SD* = 8.32) experience with 95% working in the field of autism therapy or support, mostly with children aged 0–12 (65%). Within the full sample, the ATI-S mean score representing the participants’ *affinity towards technology* was 3.97 (*SD* = 0.94) indicating a medium to high affinity towards technology on the 6-point Likert scale with higher scores indicating higher affinity. Within groups, mean scores differed significantly (autistic adults: *M* = 4.32, *SD* = 0.89, professionals: *M* = 3.58, *SD* = 0.84; caregivers: *M* = 4.02, *SD* = 0.94; *p* < .01). Around half of the caregivers and half of the autism professionals reported that they have already used autism-specific assistive technologies, in contrast to only 23% of the adults on the AS.

**Table 1:** Sociodemographic information of the total sample by group.

Variable	Adults on the AS		Autism professionals		Caregivers	
	*N* = 40		*N* = 40		*N* = 43	
	M	SD	M	SD	M	SD
Age	38.42	12.36	40.83	10.46	45.98	6.11
Children’s age (caregiver)	–	–	–	–	12.79	5.66
Affinity for technology (ATI-S)	4.32	0.89	3.58	0.84	4.02	0.94
	N	%	N	%	N	%
Females	26	65%	28	70%	35	81%
Males	13	33%	11	28%	8	19%
Non-Binary	1	2%	1	2%	0	0%
Education^a^						
Lower secondary education	8	20%	0	0%	7	16%
Upper secondary education	10	25%	4	10%	4	9%
Academic education	20	50%	36	90%	32	74%
Use of autism-specific assistive technologies						
No	31	76%	18	45%	21	49%
Yes	9	23%	22	55%	22	51%
Age group working with						
0–12 years	–	–	26	65%	–	–
13–21 years	–	–	10	25%	–	–
> 21 years	–	–	4	10%	–	–
Specialization						
Autism therapy or support	–	–	31	76%	–	–
Autism diagnostics	–	–	0	0%	–	–
Both	–	–	7	18%	–	–
Other	–	–	2	5%	–	–
Children’s intelligence level						
IQ ≤ 85	–	–	13	33%	14	33%
IQ > 85	–	–	27	68%	26	61%

*AS* autism spectrum, *ATI-S* Affinity for technology interaction short scale, *IQ* intelligence quotient.

^a^The level of education was determined according to the International Standard Classification of Education (ISCED) applied to the German school system: Lower secondary education (ISCED-Level-2); Upper secondary education (ISCED Level-3); Academic education (ISCED-Level- 6/7). All percentages have been rounded.

### Global acceptance and intention to use RAT

The group of stakeholders with high *global acceptance* scores (ratings > 60 on a scale ranging from 0 to 100) regarding RAT (*N* = 72; 59%) was significantly larger (χ^2^(1) = 3.58, *p* = .03) than the group with low to moderate acceptance scores (*N* = 51; 41%) (H1). This was also true for intention to use. Here, the group with high scores on intention to use (*N* = 78; 63%) was significantly larger (χ^2^(1) = 8.85, *p* < .01) than the group with low to moderate acceptance scores (*N* = 45; 37%).

### Comparison of technologies and groups

**Fig. 2:**
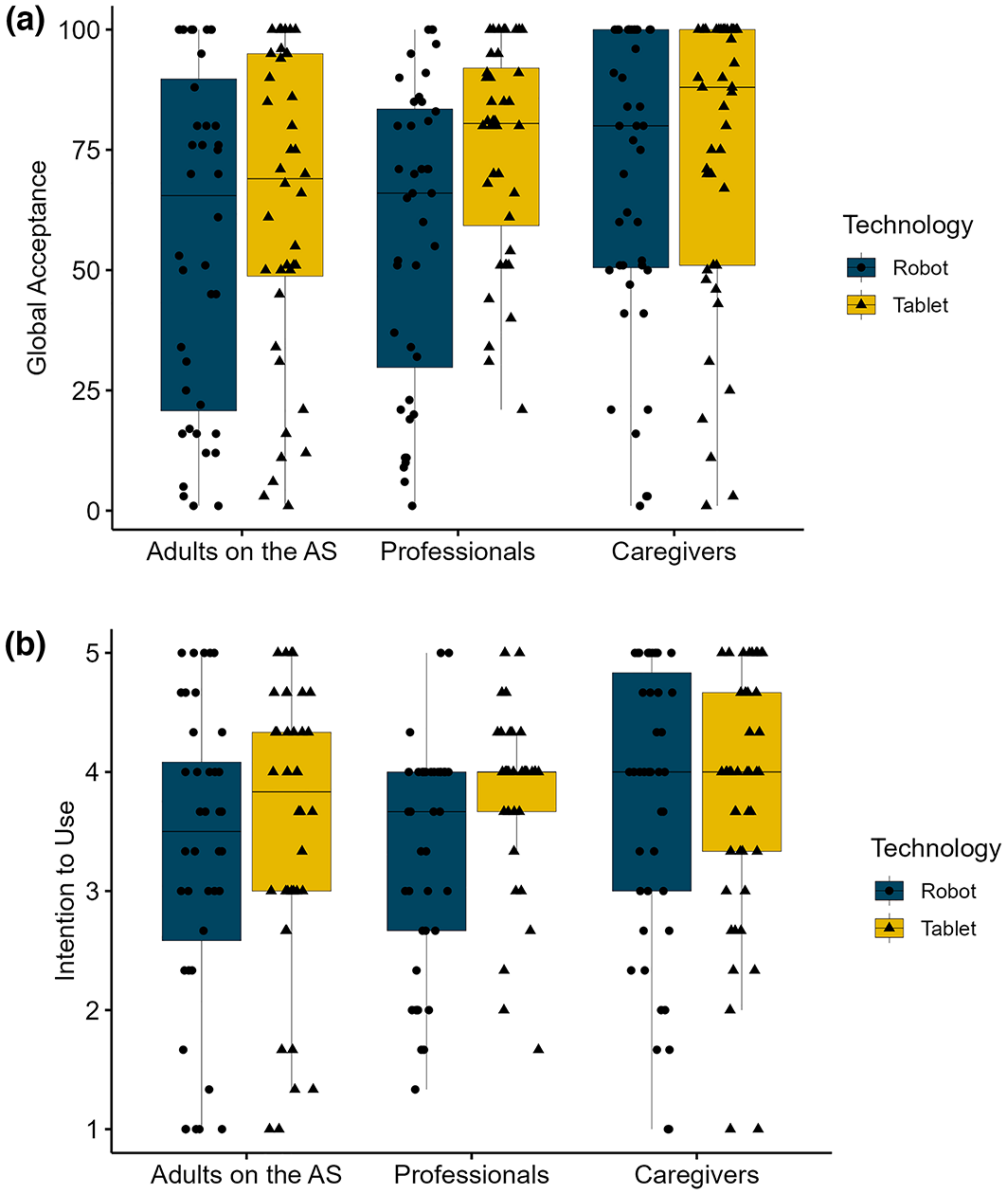
Boxplots representing the results of each stakeholder group for (**a**) *Global Acceptance* and (**b**) *Intention to Use*. Center line represents median; lower and upper box limits represent quartiles 1 and 3. Whiskers represent 1.5 interquartile range. *Note*: *AS* autism spectrum.

Results of the ALMERE questionnaire and a two-way mixed MANOVA are reported in Table 2. Overall, analyses showed a statistically significant difference between the technologies on the combined dependent variables, *F*(8, 113) = 9.63, *p* < .01, Wilk’s Λ = 0.60 (H2). Regarding *global acceptance*, a two-way mixed model ANOVA with technology as within-subjects factor and stakeholder group as between-subjects factor (Fig. 2a) showed a significant main effect of technology, *F* (1, 120) = 17.03, *p* < .01, η_G_^2^ = 0.03, with TBT (*M* = 71.20, *SD* = 28.87) having higher acceptance levels than RAT (*M* = 61.60, *SD* = 32.90). No significant main effect of group (*F* (2, 120) = 1.85, *p* = .16, η_G_^2^ = 0.03) nor an interaction effect was detected (*F* (2, 120) = 2.85, *p* = .62, η_G_^2^ < 0.01), implying that differences in the acceptance ratings for both technologies did not vary between stakeholder groups.

**Table 2:** Results of the seven subscales on acceptance-related factors assessed by the ALMERE questionnaire and MANOVA comparisons.

	RAT		TBT		Results of the ANOVA, effects of		
	M	SD	M	SD	Technology	Group	Group x Technology
Intention to use	3.46	1.16	3.73	0.99	***F*(1**,** 120) = 12.10**, ***p*** **< .01**,** η**_**G**_^**2**^ **= 0.02**	*F*(2, 120) = 1.32, *p* = .27, η_G_^2^ = 0.02	***F*(2**,** 120) = 3.37**, ***p*** **= .04**,** η**_**G**_^**2**^ **= 0.01**
Perceived usefulness	3.60	1.00	3.90	0.84	***F*(1**,** 120) = 18.20**, ***p*** **< .01**,** η**_**G**_^**2**^ **= 0.03**	*F*(2, 120) = 0.32, *p* = .73, η_G_^2^ < 0.01	*F*(2, 120) = 1.96, *p* = .15, η_G_^2^ < 0.01
Perceived ease of use	3.62	0.79	3.97	0.68	***F*(1**,** 120) = 34.21**, ***p*** **< .01**,** η**_**G**_^**2**^ **= 0.06**	*F*(2, 120) = 2.15, *p* = .12, η_G_^2^ = 0.03	*F*(2, 120) = 2.39, *p* = .96, η_G_^2^ < 0.01
Social influence	3.31	0.92	3.68	0.76	***F*(1**,** 120) = 26.56**, ***p*** **< .01**,** η**_**G**_^**2**^ **= 0.05**	*F*(2, 120) = 1.28, *p* = .28, η_G_^2^ = 0.02	*F*(2, 120) = 2.04, *p* = .14, η_G_^2^ = 0.01
Attitude	3.65	1.01	3.86	0.87	***F*(1**,** 120) = 8.78**, ***p*** **< .01**,** η**_**G**_^**2**^ **= 0.01**	*F*(2, 120) = 0.61, *p* = .54, η_G_^2^ = 0.01	*F*(2, 120) = 1.04, *p* = .36, η_G_^2^ < 0.01
Perceived enjoyment	3.84	0.86	3.80	0.79	*F*(1, 120) = 0.25, *p* = .62, η_G_^2^ < 0.01	*F*(2, 120) = 1.36, *p* = .62, η_G_^2^ < 0.01	*F*(2, 120) = 1.14, *p* = .32, η_G_^2^ < 0.01
Facilitating conditions	3.81	0.95	4.15	0.73	***F*(1**,** 120) = 17.46**, ***p*** **< .01**,** η**_**G**_^**2**^ **= 0.04**	*F*(2, 120) = 2.14, *p* = .12, η_G_^2^ = 0.03	*F*(2, 120) = 0.90, *p* = .41, η_G_^2^ < 0.01

Means scores (*M*), standard deviations (*SD*) within condition (RAT/TBT), and main effects of technology and group, group X technology interaction.

*RAT* robot-assisted training, *TBT* tablet-based training, *df* degrees of freedom, *η*_*G*_^2^ effect size, eta squared, *F* F statistic, *M* Mean, *p* significance level, *SD* standard deviation, Significant values are in bold.

Results for all subscales of the ALMERE questionnaire are summarized in Table 2. For the subscale *intention to use* (Fig. 2b), a significant main effect of technology was detected (*F* (1, 120) = 12.10, *p* < .01, η_G_^2^ = 0.02) with participants being more willing to employ TBT (*M* = 3.73, *SD* = 0.99) than RAT (*M* = 3.46, *SD* = 1.16). While no effect of the stakeholder group was detected (*F*(2, 120) = 1.32, *p* = .27, η_G_^2^ = 0.02), a significant interaction between technology and group was indicated (*F*(2, 120) = 3.37, *p* = .04, η_G_^2^ = 0.01), primarily driven by differences in the group of autism professionals, where TBT (*M* = 3.84, *SD* = 0.70) received significantly higher *intention to use* ratings than RAT (*M* = 3.32, *SD* = 0.95). Concerning the subscales *perceived usefulness*, *perceived ease of use*, *social influence*, *attitude towards technology*, and *facilitating conditions*, a significant main effect of technology was detected for each subscale with the TBT-approach consistently receiving higher ratings than the RAT-approach. This was not the case for the subscale *perceived enjoyment*, which showed no significant differences between the acceptance ratings for RAT and TBT (*F*(1, 120) = 0.25, *p* = .62, η_G_^2^ < 0.01).

Comparing the two technologies, 35% of stakeholders indicated a preference for the TBT-approach, ~ 25% preferred the RAT-approach, and 33% reported supporting both approaches. A minority (~ 7%) rejected the use of both scenarios.

### Primary factors affecting intention to use

The multiple regression analysis aimed at predicting *intention to use* by the factors *perceived usefulness* and *perceived ease of use*, controlling for *affinity for technology* (ATI-S) was significant, *F*(5,117) = 132.20, *p* < .01, with an adjusted *R-squared* of 0.84. *Intention to use* was predicted significantly by *perceived usefulness* (*r* = .90, *p* < .01) and *perceived ease of use* (*r* = .20, *p* = .02). Although significant (*p* = .01), the factor a*ffinity for technology* only had a small influence on the *intention to use* (*r* = .13).

### Stakeholders’ concerns

**Fig. 3:**
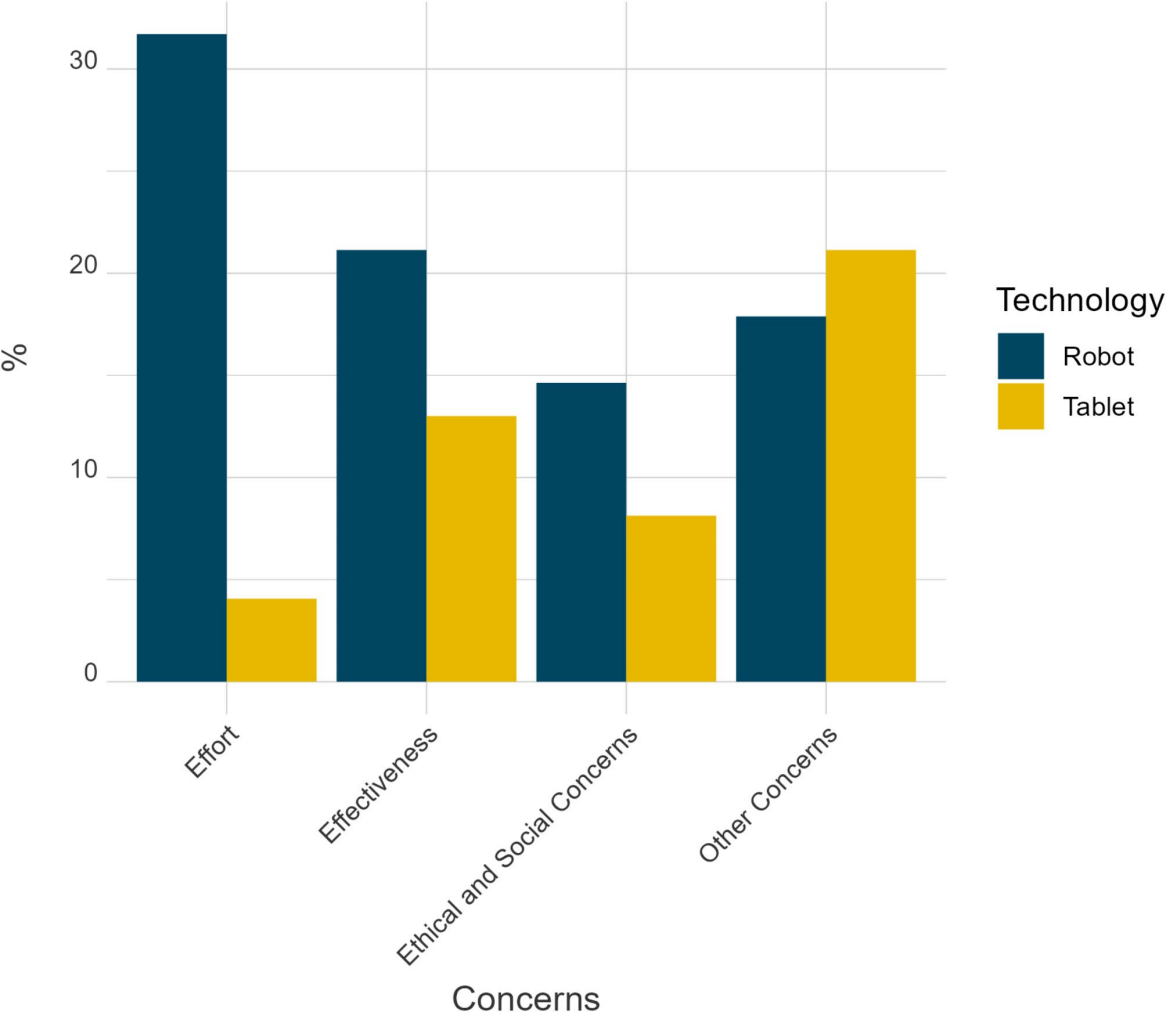
Relative proportion of concerns expressed regarding both approaches across all stakeholder groups.

Before analyzing the data on concerns quantitatively, answers given within the free text field were categorized and integrated into the given categories, when applicable. Detailed results are presented in Table 3; Fig. 3. Within the total sample, more participants expressed concerns concerning RAT (71%) than TBT (46%). Around 32% of the participants were concerned that too much effort is required to deploy the robot in the therapeutic setting, while only ~ 4% expressed these concerns regarding TBT. 21% of the participants were skeptical regarding RAT effectiveness, and 13% expressed this concern concerning TBT. Ethical concerns were raised by ~ 15% of the participants about RAT, versus ~ 8% about TBT. Other concerns (e.g., adaptability) concerning RAT were expressed by ~ 18% of the participants and by ~ 21% for TBT.

**Table 3:** Relative frequencies of expressed concerns for the full sample and within stakeholder group.

Concerns	Overall (%)		Adults on the AS (%)		Autism professionals (%)		Caregivers (%)	
	*N* = 123		*N* = 40		*N* = 40		*N* = 43	
	RAT	TBT	RAT	TBT	RAT	TBT	RAT	TBT
Effectiveness	21.11	13.01	27.50	20.00	20.00	7.50	16.28	11.63
Effort	31.71	4.07	27.50	5.00	30.00	0.00	37.21	6.98
Ethical concerns	14.63	8.13	20.00	15.00	15.00	5.00	9.30	4.65
Other concerns	17.89	21.14	25.00	27.50	12.50	20.00	16.28	16.28

*AS* autism spectrum, *RAT* robot-assisted training, *TBT* tablet-based training.

## Discussion

Our study aimed to provide a clearer understanding of acceptance levels regarding robot-assisted therapies (RAT) for autistic children. We evaluated an elaborated RAT-approach focusing on caregivers of children on the AS, autism professionals, and adults on the AS, groups rarely integrated into studies so far^32^. A tablet-based therapy (TBT) approach was implemented as a control condition to improve result interpretability. Consistent with our first hypothesis (H1), a higher proportion of the participants showed medium to high as compared to low *global acceptance* ratings for the RAT-scenario. As per our second hypothesis (H2), the ALMERE-acceptance ratings of RAT differed from TBT, with TBT being preferred over RAT across all variables (except *perceived enjoyment*) and groups.

When considering the ratings on *global acceptance* and the behavioral aspect of acceptance (*intention to use)*, stakeholders were more likely to approve than disapprove of the presented RAT-approach as indicated by the larger proportion of participants showing high, than medium to low ratings. Additionally, the direct comparison revealed a rather positive attitude towards RAT, since more than half of the participants either preferred the RAT-approach or supported both approaches. However, with considerable variability in the ratings on *global acceptance* and *intention to use*, and the majority of voiced concerns related to RAT, our findings indicate mixed acceptance of RAT, similar to other studies^25,32,41,42^.

Importantly, our study adds a more differentiated perspective comparing the findings of RAT acceptance with those of a TBT-approach. Our results indicate a clear, although small, preference for TBT. This is further supported by a larger approval rate for TBT versus RAT in the direct comparison. Similar findings were reported by one of the few controlled studies evaluating parents’ acceptability of RAT for non-autistic children with oppositional behavior in comparison to an internet-based approach and a waitlist-control condition^38^. While both technology-based interventions were largely accepted, parents preferred the more familiar internet-based intervention approach^38^. In contrast, numerous studies in the field of human-robot interaction with non-autistic, mostly adult participants reported several advantages of embodied robots over virtual avatars (e.g., being more favorable/social^60,61^, enhancing compliance^62^, or increasing engagement^63,64^). Concerning child-robot interactions in health care settings, Jeong, Breazeal, Logan & Weinstock^65^ reported that the Huggable robot, designed to promote social-emotional well-being, was more accepted than its corresponding virtual avatar.

However, these human-robot interaction studies are only comparable to a limited extent to our study. While participants in human-robot interaction studies typically engage in real interaction with a robot, we used an online video presentation. Thus, our setting is missing the feeling of the robot being an embodied agent, which could contribute to an interaction being perceived as more natural, intuitive, and physical^66^. In a real-life demonstration of a child-robot interaction, stakeholders might directly observe the positive effects of the robot on the children’s behavior^67,68^ and well-being^15,17,47^. This might have also resulted in TBT being anticipated as being equally enjoyable for children, which contrasts with the findings mentioned above^60,61^. Nevertheless, the general suitability of robots, as a motivational tool for engaging children on the AS in therapy (compare e.g^69^). could be confirmed.

Moreover, we aimed to investigate the acceptability of RAT in a realistic application context and among potential future users. Here, application-oriented factors such as *perceived usefulness* or *perceived ease of use*might be more important than other aspects of the robot such as its pleasantness. Indeed, within the evaluations of the acceptance-related factors targeted by the ALMERE model^22^, the general preference for TBT was further emphasized. Stakeholders perceived the TBT to be generally more useful (*perceived usefulness*) and usage of the robot in therapy was expected to be significantly less easy than that of the tablet (*perceived ease of use*). In the conducted regression analysis study, we found especially *perceived usefulness* to be an important factor in predicting intention to use/support the use of RAT within autism therapy. Since current RAT development has the overall goal of implementing social robots in children’s real-life therapy or support, it is crucial to understand the reasons for these findings.

We might speculate that the comparably lower *perceived usefulness*of the robot might have resulted from the specific RAT-scenario presented. While the robot’s embodiment might have been perceived as being essential for the emotion regulation module (especially during the robot-guided breathing exercises), participants might not have judged the robot to add extra value to the facial emotion recognition module. This could have lowered its perceived usefulness. Future studies should therefore carefully investigate which therapy scenarios in particular might profit from using robots. Regarding this aspect, tablets are a far more established technology, thus, most of the participants might have experience in their use^38^. This fact might also have lowered the *perceived usefulness* and *ease of use* of the robot. Similarly, the therapeutic capabilities and effectiveness of digital training or digital health applications were largely investigated and reported in the last decades^70,71^, resulting in increased public information and usage. In contrast, robots represent a much more unknown technology, which is rarely represented in public life. Hence, most participants might have never used, or even seen, a social robot. This uncertainty might have induced feelings of anxiety, which, in turn, decreased *perceived usefulness* and *ease of use* ratings as postulated by the ALMERE model^22^. Additionally, the clinical effectiveness of social robots has been only rudimentary investigated so far^16^. Thus, the public might not be informed well about the clinical potential of robots. Indeed, in the present study, concerns about the effectiveness of the approach were expressed with considerably greater frequency with regard to RAT than to TBT. In contrast to previous studies^42,43^, we did not find the professionals reporting to be skeptical that they have the skills necessary to use the robot in a meaningful way. Nevertheless, participants estimated that the robot would be significantly more difficult to implement in existing therapeutic settings than the tablet, as shown by ratings on the “facilitating conditions” subscale of the ALMERE questionnaire. This might relate to the main concerns expressed in our study, stating that much effort might be required to deploy the robot in therapeutic settings and that the costs of RAT might be too high. These concerns were expressed by approximately one-third of participants (compare^41–43^), while the TBT-approach evoked similar concerns in only a minority. This confirms previous findings^40^ that perceived cost-benefit considerations play an essential role in RAT acceptance.

Regarding the factors, *attitudes towards technology* and *social influence*, results showed that participants, on average, expressed substantially more positive attitudes towards the TBT-scenario than towards RAT and anticipated that other persons would evaluate the TBT-approach more favorably than RAT. Moreover, our results show that a considerably higher number of participants expressed ethical and social concerns regarding RAT than regarding TBT. Since ethical aspects have a considerable influence on user acceptance^52,72,73^, it is reasonable to assume that this has also had an impact on overall acceptance as well as ALMERE subscales in the present study.

The findings of this study permit several implications to be drawn. Overall, future research and development should aim to result in RAT solutions that are proven to be effective, perceived to be useful and easy to use, and thus, highly accepted by future users and stakeholders. The factors discussed previously represent important starting points. In this context, studies that examine easy and cost-effective ways in which robots can be integrated into autism support/therapy while respecting the existing intervention protocols and settings are highly needed^15,69^.

Additionally, informing more explicitly about how potential ethical and social challenges (e.g., the child developing an emotional bond to the robot^31,74^) could be prevented, might, in turn, enhance stakeholders’ acceptance ratings. To ensure the safe usage of RAT, ethical, legal, and social concerns as well as therapeutic considerations should be further investigated (see^31,75^ for one of the first surveys) to provide a solid foundation for safe RAT development.

Finally, targeting the stakeholders’ attitudes, experiences, and expertise already within RAT development might enhance its later acceptance and usage^76,77^. User-centered development might further ensure that essential principles such as self-determination and inclusion are not compromised^19,32,63,69^. Since the individual stakeholders in the present study varied largely in their ratings and, additionally, the group of autism professionals seems to be less willing to use RAT, studies should include different groups of stakeholders in the development and research process to allow distinct insights and different perspectives^32^.

Importantly, the perspectives of individuals on the AS should be integrated. In case autistic children cannot be consulted themselves (e.g., due to their developmental level), adults on the AS might provide essential personal insight^78^, providing informed perspectives, which might differ from the opinions of other stakeholders. Indeed, as shown descriptively, the adults on the AS expressed more concerns towards RAT (and TBT) than the two other groups, especially regarding ethical and social aspects and the expected effectivity). On the other hand, the lack of differences in acceptance ratings between groups and the considerably high acceptance ratings of RAT also in adults on the AS are promising. In sum, our results underline the importance of assessing the attitudes and perspectives of different stakeholder groups, and their potential differences, to gain differentiated and holistic perspectives on the application of RAT for children on the AS.

Several limitations of this study should be acknowledged, pointing to directions for future research. While the implementation of this study in the form of an online survey has advantages such as easily allowing to integrate comparatively large samples, also within clinical populations, it is also the source of many limitations. First, compared to laboratory experiments, the inherently less controllable research setting may have had an impact on the high level of variance. Second, the sample might not have been representative of the underlying population, as internet-based surveys are more likely to recruit technologically affine participants, resulting in an overestimation of the stakeholders’ acceptance of RAT. However, the participants’ general affinity for technology (ATI-S) was found to be only weakly associated with ratings on intention to use (see regression analysis). Although the affinity for technology was medium to high, the ratings on the ATI-S still varied between very low and very high. In addition, a fraction of participants expressed a general skepticism towards using technology in autism therapy (included in “other concerns”, Table 3). These findings indicate that not only high technology affine persons have been included and that our findings are meaningful in terms of the acceptance of RAT in stakeholder groups.

This study focuses on comparing two technology-based approaches, RAT and TBT, as emerging and established tools in therapeutic contexts. This comparison provides valuable insight into the unique characteristics and challenges of RAT as a novel intervention, utilizing TBT as a benchmark for stakeholder acceptance. While the additional inclusion of a human-based training condition could have provided further valuable insights, it would have significantly increased the complexity of the study, particularly in terms of time demands on the subjects. Although it was therefore beyond the scope of the current research, future studies could extend the current findings by including human-based conditions to provide broader contextual insights.

The biggest limitation, however, is the lack of interaction with an actual embodied robot due to the online presentations of the RAT/ TBT-scenarios. As already mentioned in the previous sections, the physical interaction with a robot has a large impact on users, especially during first encounters^42,79,80^ and further, differences between the two approaches may be more difficult to discern based on text and video-based information. Hence, the observed preference for TBT might be partly due to the used methodology. While the effectiveness of the developed scenario was subsequently analyzed in an HRI study (the results of which will be published in a future article), further research is needed to validate both the effectiveness as well as acceptance-related results in a real interaction setting.

Finally, two specific technology-based approaches have been compared in this study, which limits the generalizability of the results concerning the broader acceptance of RAT across the Autism Spectrum. The training scenarios were specifically tailored for autistic children without co-occurring intellectual difficulties, addressing the needs of this particular subgroup within the spectrum. However, this focus may have influenced how stakeholders evaluated the interventions, particularly if their experiences or expectations extended to other sub-groups with differing levels of functioning or needs. Nonetheless, since the RAT scenario was developed by clinical psychologists with extensive experience in autism support, it represents a realistic and clinically relevant example of potential integration. These findings can therefore be interpreted as reflecting a positive acceptance of RAT by stakeholders within the defined context of this study, while highlighting the need for future research to explore its acceptance across a more diverse range of AS profiles.

In conclusion, robots in general and therapeutic robots, in particular, represent a topic of much controversy. In this context, the present study underscores the importance of stakeholder involvement to ensure that RAT-approaches meet the needs of those affected, here, children on the AS. Further, the study demonstrates the importance of implementing a relevant control condition for the analysis of the acceptance assessments.

Overall, the results are promising for future usage of robots within autism therapy since a medium to high global acceptance of RAT and stakeholders’ intention to use RAT was indicated. Nevertheless, considerable variability in the ratings was observed, indicating controversial opinions among participants. The slight preference towards TBT might be due to tablets representing the more established technology and the lack of stakeholders’ experience with robots, which was not targeted sufficiently due to the online presentation of the approaches. Therefore, the present study should be replicated, using a real-life demonstration of both conditions. Future RAT development should aim to improve effectiveness and ease of use, reduce deployment effort, and target the stakeholders’ ethical and social concerns, e.g., by implementing user-centered development that integrates stakeholders’ perspectives. Finally, feasibility studies with technology-based control conditions should investigate the specific conditions that allow to create an actual clinical benefit of RAT for children on the AS and other stakeholders.

## Methods

### Study design and procedure

**Fig. 4:**
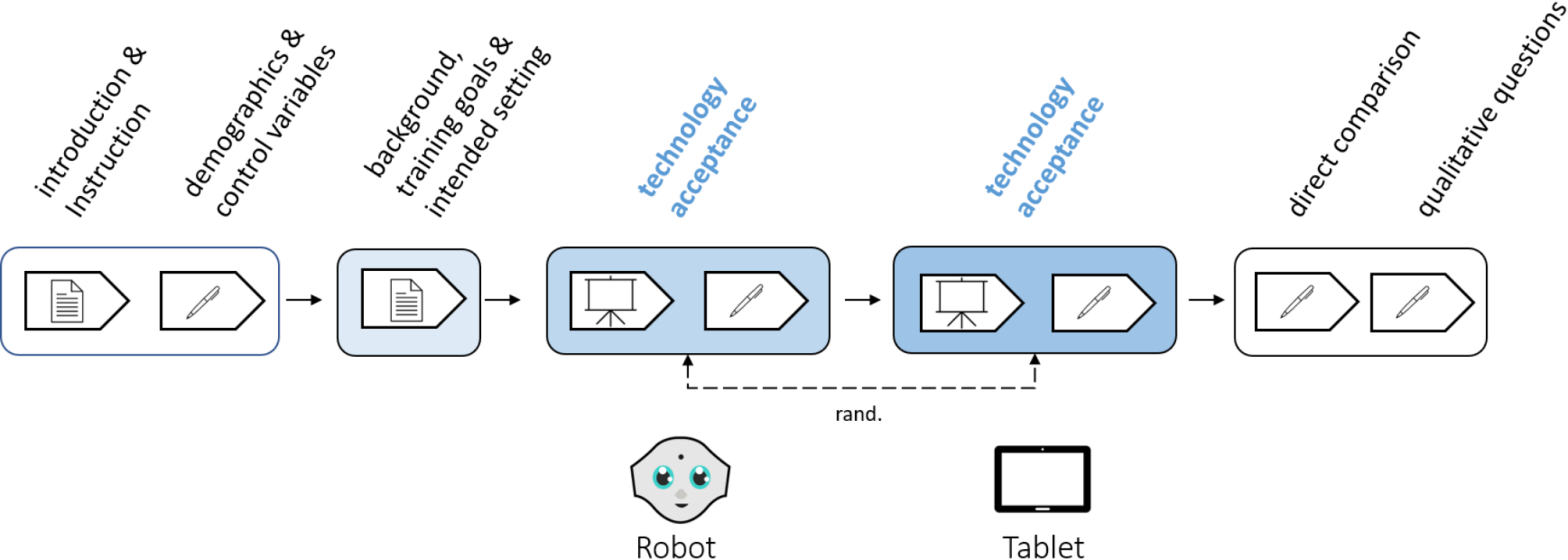
Study Design. After instruction and collection of relevant control variables, participants were given background information on the training scenarios developed, as well as the training setting and context. Subsequently, both the RAT and the TBT-scenario were presented via video. Acceptance of the technology was measured immediately after each video. The order of the two scenarios was randomized between participants. Finally, a direct comparison of the technologies was made and possible concerns were collected.

Due to Covid-related restrictions at the time, the study was conducted as a 30-minute video-based online survey. After undergoing the informed consent procedure and a general introduction to the study, sociodemographic data were obtained and *general affinity towards technology* (*Affinity for Technology Interaction Short Scale*; ATI-S^81^) and the participants’ experiences with autism-specific assistive technologies (single item) were assessed to control potential influencing factors on the acceptance ratings. The evaluation of the acceptance of RAT in comparison to TBT was realized in a within-subject study design (Fig. 4), in which each participant assessed both, a specific RAT-scenario for children on the AS (Block A) and a TBT control scenario (Block B). Within each block, participants received background information about the respective scenario, followed by a demonstration video explaining the RAT (Fig. 1a), or, respectively, the TBT-approach (Fig. 1b). The order of the presentation of the two blocks was randomized across participants. After the presentation of each demo video, the acceptance of the previously presented approach (RAT/TBT) was measured by a single item on *global acceptance* and the adapted ALMERE questionnaire assessing the *intention to use*the novel approach and further acceptance-related factors^22^. When the evaluation of both approaches was completed, the participants were requested to compare the two approaches directly and to answer qualitative questions addressing concerns associated with the RAT and TBT-approaches. For compensation, all participants were entered into a lottery for winning 10 x €15 as compensation. The study was performed in line with the principles of the Declaration of Helsinki and received approval from the Ethics Committee of the Humboldt Universität zu Berlin (number 2021-14). Informed consent was obtained from all participants prior to participation.

### RAT-scenario

The RAT-scenario (Fig. 1a) was developed in the joint research project ERIK by a multidisciplinary team with backgrounds in clinical psychology, medical engineering, robotics, and other disciplines. The approach aimed at developing an emotionally sensitive robotic platform for the training of socio-emotional competencies in autistic children aged 7–12 years without intellectual disabilities^71,72^ (see https://www.youtube.com/watch?v=R9Tr9Qe0K2M for an online-demonstration of the project and the developed RAT-approach with English subtitles).

The humanoid robot Pepper (Softbank Robotics) was implemented with the function of a tutor and motivator to foster emotion recognition (module I) and emotion regulation (module II). The two robot-assisted training modules are presented on the robot’s integrated tablet screen. In module I, the robot engages the child in a learning game to practice recognizing five basic emotions and a neutral state based on video examples of facial expressions. In module II, the child learns to deal with high and negative arousal, thus with stress and frustration. The emotions were induced via a video game presented and moderated by the robot, while the robot regularly guided the child through breathing exercises in order to facilitate emotion regulation. Following focus group interviews and workshops with relevant stakeholders, the developed intervention was designed takes place in a triadic interaction between the robot, the child, and the therapist. Within the robot-child interaction, the robot operates semi-autonomously. Thus, although an automatically and independently running program determines the behavior of the robot, a present human therapist remains in supervisory control and, if necessary, adapts the interaction to the child’s needs by using an external control tablet (e.g., the robot might suggest a break or a calming exercise if the child appears distressed, see^56^).

To support the therapist’s decisions, the external control tablet al.so provides the therapist with real-time information on the child’s current inner state. For this purpose, the system records and automatically interprets physiological data such as the children’s heart rate (SHORE, Fraunhofer IIS^73^), facial expressions (SHORE, Fraunhofer IIS), and tone of voice (AudEERING) employing its built-in cameras and microphones.

### Presentation of the RAT and TBT conditions in the present study

Both conditions (RAT/TBT) were presented by two three-minute demonstration videos. The videos demonstrated the approaches from a first-person perspective so that participants assumed the child’s position within a potential child-technology interaction scenario. Both videos were embedded into background information on the respective approach (intervention goals, setting, technology, and emotion-sensitive technology) through a combination of explanatory texts and pictures. In the demonstration videos introducing the tablet-based control condition (TBT), the physical robot Pepper was replaced by a similarly looking virtual robot avatar, which is presented on a tablet screen (see Fig. 1b). The setting and goals of the TBT-approach were similar to the RAT condition. Both videos are available in German language: RAT: https://youtu.be/jHW6QCb5H7o, TBT: https://youtu.be/6o_uthq8K-Y)

### Participants

Inclusion criteria for autism professionals were a principal occupation in diagnosis, therapy, education, or support of autistic individuals with a professional experience of at least one year. Adults on the AS were eligible, if they indicated a clinical diagnosis of autism spectrum disorder (ICD-10: F84.0, F84.1, or F84.5). Caregivers were included when they had at least one child aged 29 years or younger with clinically diagnosed autism (ICD-10: F84.0, F84.1, or F84.5). This age cutoff was established to ensure that memories of their children being within the target age range (7–12 years) are relatively recent and relevant to the study. An a priori power analysis (WebPower package R 4.1.0^82^) revealed a minimum sample size of *N* = 111 for detecting an assumed medium effect size of *f* = 0.3 by conducting a mixed ANOVA with three groups, two measurements, a test power of 80% (1-β), and a significance level of α = 0.05. In total, 136 participants were recruited through various autism institutions (e.g., outpatient clinics, autism care units), associations of parents and affected persons (e.g., Aspies e.V., Autismus Deutschland e.V.), social-pediatric centers, and through various internet forums. Due to not meeting the inclusion criteria, 13 participants had to be excluded from further analysis, resulting in *N* = 123 valid data sets. The stakeholder groups were comparable in size, with *N* = 40 adults on the AS, *N* = 40 autism professionals, and *N* = 43 caregivers of autistic children.

### Measures

#### Global acceptance

The stakeholder’s global acceptance of RAT and TBT-scenarios was measured by using the single item „*How strongly would you approve the presented robot-/tablet-assisted approach being used to support autistic children*?”, which was rated on a sliding scale ranging from 0 to 100% (“*I approve … %*”).

#### Intention to use and acceptance-related factors

The used technology acceptance questionnaire was based on the ALMERE model^22^. For the present study, the ALMERE questionnaire was adapted in wording to the autism-specific application context. Subscales that could not be answered meaningfully based on the provided online information and setting were excluded (e.g., the subscales *anxiety* or *trust* demand a previous real interaction with the technology). The resulting 22 items targeted seven constructs measured on seven subscales *(Intention to Use*, *Perceived Ease of Use*, *Perceived Usefulness*, *Facilitating Conditions*, *Perceived Enjoyment*, *Attitude towards Technology*, *Social Influence*, see Table 4).

**Table 4:** Acceptance questionnaire adapted from the ALMERE questionnaire^22^.

ALMERE subscale	Original definition	Targeted aspect	Items (selected examples)
Intention to use	The outspoken intention to use the system over a longer period.	Intention to use the RAT/ TBT-approach in autism therapy (professionals)/intention to support the use in autism therapy (adults on the AS, caregivers)	I would like to see the [RAT/TBT] approach used in the future.
Perceived usefulness	The degree to which a person believes that using the system would enhance his or her daily activities.	Extend to which the RAT/ TBT-approach is believed to be effective concerning the intervention goals.	I think the [RAT/TBT] approach would support autistic children in many ways.
Perceived ease of use	The degree to which the user believes that using the system would be free of effort.	Expected effort related to utilizing the robot/tablet in the therapeutic context.	I think that the [RAT/TBT] approach would be easy to use to support autistic children.
Social influence	The user’s perception of how people who are important to them think about him/her using the system.	Anticipation of the attitude of other people towards the RAT/ TBT-approach.	I think many people would like to see the [RAT/TBT] approach used to help autistic children.
Attitude	Positive or negative feelings about the application of the technology.	Sentiment- and emotion-related aspects of acceptance ratings of RAT/TBT.	I think that it is a good idea to use the [RAT/TBT] approach to support autistic children.
Perceived enjoyment	Feelings of joy or pleasure by the user are associated with the use of the system.	Pleasure or joy is attributed to the autistic children feel during the course of the intervention.	I think that autistic children would find the [RAT/TBT] approach entertaining.
Facilitating conditions	Objective factors in the environment that facilitate using the system.	The degree to which the RAT/ TBT-approach is perceived to be integrated easily into standard autism therapy.	I think that the [RAT/TBT] approach is well compatible with other existing approaches.

Subscales, original definition by Heerink et al.^22^, targeted aspects in the present study, and examples of items.

*AS* autism spectrum, *RAT* robot-assisted therapy, *TBT* tablet-based therapy.

#### Direct comparison of RAT versus TBT

For direct comparison, participants were asked which scenario they would support to be applied in autism therapy for children. Responses to the single-choice-item were given by selecting one of four options: “*RAT*”, “*TBT*”, “*Both of them*”, and “*None of them*”.

#### Assessment of control variables

The German version of the Affinity for Technology Interaction Short Scale (ATI-S^81^) was administered to control for a possible influence of the participants’ general affinity towards technology on their acceptance ratings. The questionnaire consists of nine items with answers being given on a 6-point Likert scale (“*completely disagree*” – “*completely agree*”). Additionally, the use of autism-specific assistive technologies was accessed by using a single-item question.

#### Qualitative assessment of stakeholders’ concerns

The participants’ concerns regarding RAT and TBT were assessed by using a single-item question asking the participants to indicate their possible concerns by selecting either (1) concerns regarding effectiveness, (2) concerns regarding the assumed effort, and (3) ethical concerns. Other concerns could be expressed by using a free text field.

### Statistical analysis

On all acceptance measures and the ATI-S, numerous outliers (values outside +/- 3 SD) were identified, especially in the group of autistic adults. Although removing the outliers might have counteracted the differences in variance, they were included in the further analysis because there was no evidence that the deviations were due to measurement errors and large divergences in the assessment of new therapeutic approaches are plausible. Differences between the individual stakeholder groups in sociodemographic variables and affinity for technology (ATI-S) were tested by using a single factor ANOVA, or, respectively, a χ^2^ -test for categorical variables.

A multivariate analysis of variance (MANOVA) was conducted for all acceptance variables (global acceptance, 7 ALMERE subscales) with technology as a within-subjects factor and group as a between-subjects factor. In addition, a two-way mixed-design analysis of variance (ANOVA) was conducted for each variable (global acceptance, 7 ALMERE subscales). The assumptions for conducting an ANOVA were checked for each variable by using the Shapiro-Wilk test of normality^83^, the Box’s M test^84^ for testing homogeneity of covariances, and the Levene test^85^for checking the homogeneity of variances. Since the tests showed that some assumptions were violated, the non-parametric generalized Kruskal-Wallis-Friedman-Test, also known as Puri & Sen-Test^86–88^ was applied in addition to the mixed ANOVA. Since both tests reached the same conclusions across all tested outcome variables, and given that F-statistics can be expected to be robust as *type I error*rates are well controlled provided sufficiently large sample sizes and groups of equal size^89–92^, the results of the mixed ANOVAs are reported. Pairwise comparisons of group means were performed using a Games-Howell-Test^93^ with appropriate adjustment for multiple testing (*Bonferroni*). Additionally, a between-subject comparison between the acceptance ratings of the first block of the experiment was performed as a two-way ANOVA with independent measurements to ensure that potential differences in ratings between the two presented therapeutic approaches were not provoked by the within-subject design. Finally, a regression analysis was conducted to investigate if the intention to use is predicted by the acceptance-related factors *perceived ease of use*, *perceived usefulness*, and general *affinity for technology*. All statistical analyses were performed using R^82^, and figures were produced using the package ggplot2^94^.

## Data Availability

The datasets generated during the current study are available from the corresponding author on reasonable request.

## Footnotes

1 The effectiveness of the developed scenario was subsequently analyzed in an HRI study. The results of this real-world interaction study will be published in a future article.
